# Revisiting the work-suicide link: renewed evidence and models of analysis in workplace contexts

**DOI:** 10.3389/fpsyg.2023.1290079

**Published:** 2023-10-19

**Authors:** José Antonio Llosa, Esteban Agulló-Tomás, Sara Menéndez-Espina, Beatriz Oliveros

**Affiliations:** ^1^Faculty of P. Ossó, Department of Social Education, University of Oviedo, Oviedo, Spain; ^2^Faculty of Pyschology, Department of Psychology, University of Oviedo, Oviedo, Spain

**Keywords:** suicide, suicidal ideation, precarious work, job insecurity, psychosocial model, prevention

## Abstract

Suicide is a priority public health problem for the World Health Organization. It is a multifactorial phenomenon, for which there is no effective strategy for prevention and reduction. The scientific knowledge generated has not paid much attention to the differentiating role of work and employment on the phenomenon of suicide. This article first presents Abrutyn’s recent conceptual model of suicide, which has a psychosocial, holistic and integrated approach. Based on this model, it examines the most recent and solid evidence and trends linking working conditions and phenomenon of suicide, identifying the most relevant findings in work stress theories. It concludes by pointing out avenues of development for a more holistic and ecological understanding of suicide.

## 1. Introduction

Suicide has become a major global public health concern (WHO, 2021). In 2019, the World Health Organization (WHO) reported that 1 in 100 deaths worldwide were caused by suicide, with men’s rate double that of women. In Europe, suicide affected 11.5 people out of every 100,000 in 2019 (WHO, 2021), increasing in recent years in mid-western and south-western regions of the continent. The same has occurred in the United States, with an increase of between 2 and 5% year-on-year since 1999 (Trust for America’s Health, 2021). This problem is particularly significant in Asian countries, with the significance reflected in the Japanese, Chinese and Korean scientific literature (Kim et al., 2017, 2022; Tsuno and Tabuchi, 2022; Lin et al., 2023).

The study of suicide in the psychological sciences has progressed along two lines that tend to be disconnected, if not incoherent. The first of these, the most developed in the scientific literature, involves exploring the phenomenon in individual terms (Rodriguez-Otero et al., 2022): from approaches that follow genetic patterns (Sokolowski and Wasserman, 2021) to cognitive and/or personality elements, as an effect of the postpartum situation (Amiri and Behnezhad, 2021) through traits such as impulsiveness (Beach et al., 2022; Brokke et al., 2022), emotional intelligence (Korkmaz et al., 2020) or cognitive rigidity (Brokke et al., 2020). Suicide, from this perspective, has been understood as a phenomenon closely linked to personal or family causes. Its preponderance implies that the preventive approach focuses on individual causes without the possibility of formulating an ecological perspective (understood as the natural context of the person) in terms of prevention (Rodriguez-Otero et al., 2022). The second perspective, which is heir to Durkheim’s (2018) understanding of suicide as a social fact, has tried to explore the social causes of a structural nature that explain the prevalence of suicide at different times and in different contexts, because of the high cultural influence that is attributed to the phenomenon (Abrutyn and Mueller, 2018; Abrutyn, 2019).

The limits of the first approach mean that suicide is inevitably a phenomenon of such complexity that to seek a theoretical explanation and a reliable measure of it is a difficult task (Large et al., 2022). This position prevents the creation of a solid contextual framework to try to understand suicide as a psychosocial phenomenon, limiting it only to a merely clinical one (Rodriguez-Otero et al., 2022). In turn, the structural perspective has tended to situate the level of analysis at which it is difficult to understand the variability between individuals with regard to this phenomenon (Abrutyn and Mueller, 2018; Mueller et al., 2021).

Abrutyn (2019), through Social Psychology, makes an effort to generate a psycho-sociological approach, which tries to reconcile both levels of analysis. He tries to renew Durkheim’s concept of deregulation, endowing it with an interconnected structural, personal and psychosocial dimension. Deregulation, in this context, means the process of losing the nexus with others, and with the contexts of relating to others (Mueller et al., 2021). Abrutyn (2019) approach goes further than Joiner’s (2005) interpersonal theory of suicide, which argues that suicidal ideation emerges in the face of circumstances that are undesirable for the person, over which they perceive no capacity for action. Joiner thus focuses on the perception of social situations (Mueller et al., 2021). However, Abrutyn’s (2019) approach allows for a holistic understanding of suicide with the capacity to explain not only a person’s individual circumstances in the face of suicide, but the way in which contextual and interpersonal elements are linked to each other.

In putting forward these approaches, the starting point should be that suicide is understood as a personal and intentional process, which takes place in a deregulated, disaffiliative framework. This results in a rupture and/or weakening of social bonds, a loss of collective identity, and a social disruption of the person. It situates the subject in what Durkheim (2018) calls a state of anomie as a consequence of disintegration, which can lead to suicidal behavior (Abrutyn and Mueller, 2018; Abrutyn, 2019; Mueller et al., 2021).

Disruption involves an interconnected process of dissolution, disjunction and dislocation. Dissolution, as the loss of bonds or anchors with others. A loss of mutual recognition. Disjunction, as the disappearance of the expectation of stability, and dislocation, as a disconnection from the physical and material space in which to develop socially (Abrutyn, 2019). This complex process of interconnectedness generates a psychosocial framework for the understanding of suicide risk (Perry and Pescosolido, 2015), which in this article allows an analysis from the perspective of the relationship between suicide and working conditions or trajectories through recent literature in the framework of psychology.

A longitudinal analysis of 84,389 suicide cases carried out in the US population concluded that 12.1% of them were directly linked to work-related issues (Peek-Asa et al., 2021). Understanding the phenomenon of suicide as multi-causal (Abrutyn and Mueller, 2018; Mueller et al., 2021), we have compiled international literature from the last 4 years that explores the relationship between suicide and precarious working conditions. In this regard, the work of Aberg et al. (2022) examines a Scandinavian working population panel data composed of almost 1.5 million workers, and controls for variables such as gender, income and mental health diagnosis for the established relationship between working conditions and suicidal behavior (fatal or non-fatal outcome). It found that the impact of working conditions on suicide remains a relevant element in isolation from other elements. Lin et al. (2023) reached similar conclusions in a Chinese population controlling for gender. In descriptive terms, in contrast to general mental health problems (Bezerra et al., 2021), suicide-related phenomena among the working population are more prevalent among middle-aged men (35–54 years) (Peek-Asa et al., 2021; Ishikawa, 2022; Tsuno and Tabuchi, 2022). The scientific literature also points to occupational sectors at higher risk: health-related professions (The Lancet, 2017; Ishikawa, 2022), construction (Tyler et al., 2022a,b), law enforcement (Genest et al., 2021), or particularly precarious sectors (Son and Lee, 2021; Greiner and Arensman, 2022).

One thing that the literature seems to indicate is that sustaining risky working conditions over time leads to a higher incidence of the phenomenon. It is not common to observe that the trigger for work-related suicide is explosive, but rather the result of an accumulation of risks (Tsuno and Tabuchi, 2022; Lin et al., 2023). There is, however, a paucity of longitudinal studies that allow for a more in-depth analysis of this issue.

With a general view of the relationship between suicide and employment, the relevant contribution of this paper is to try to draw empirical evidence from the scientific literature, the dynamics of the deregulatory process of the labor framework, and the approaches of Abrutyn’s (2019) theoretical model. The aim is to generate a coherent and integrated understanding of occupational risks for suicide, and also to explore the limits of the recent literature studying the phenomenon.

## 2. Work and suicide evidence: a psychosocial model

### 2.1. Disjunction: expectations, stress and stability

The disjunction component appeals directly to stability (Allan et al., 2021) and expectations (Agnew, 1992; Joiner, 2005). Employment is a central axis as a framework of stability for individuals, not only on a material axis, but also on a psychological and relational level (Agulló Tomás, 1998; Llosa et al., 2018). However, the dynamics of the labor market in the Western context and the framework of globalization are fundamentally changing, precarious, mobile, in what has been called a deregulated labor context (Pacheco, 2020; Cardenas del Rey and Arribas Camara, 2022; Peters, 2022). Employment rates in the European Union as a whole show that temporary employment or part-time work, as symptomatic axes of stability, have experienced a sharp increase (Agulló-Tomás et al., 2018). This is taking place in a decidedly flexible legal framework for employment (Seccareccia, 2021). This structural framework gives rise of a phenomenon that is of growing interest: job insecurity specifically, and the study of phenomena linked to job stress in general. The concept of job insecurity takes root as the fear of losing a job that one wishes to keep, and at the same time the fear of losing the status that comes with a job that one has (Greenhalgh and Rosenblatt, 1984; Hellgren et al., 1999). It is therefore a construct centered on expectations, and thus closely linked to the idea of disjunction. The scientific literature provides evidence of a direct impact of job insecurity on mental health in general terms (Llosa et al., 2018; Russo and Terraneo, 2020), and in specific terms also related to phenomena related to suicide or suicidal ideation (Milner et al., 2014; Kim et al., 2017; Howard et al., 2022).

The approach that Social Psychology has taken to the phenomenon of suicide has mainly been based on theoretical models of work-related stress, achieving relevant results of great practical applicability, as shown in the recently published meta-analysis of 22 studies (Milner et al., 2018). This directly links the knowledge that social psychology is currently generating with the idea of disjunction in Abrutyn’s (2019) model. Thus, constructs related to work-related stress models and linked to suicide such as burn-out, observed in veterinarians, have been frequently examined (Andela, 2021); the aforementioned job insecurity linked to suicidal ideation (Kim et al., 2017; Blomqvist et al., 2022), and especially to the Job Demand-Control Model (JDC) (Andela, 2021; Son and Lee, 2021; Aberg et al., 2022; Almroth et al., 2022; Nishimura et al., 2022). The JDC model shows a congruence in the results of contemporary studies in observing that jobs with high demand, but high control, are a protective factor against suicidal ideation. This has been detected in Europe with work carried out in the Swedish context (Almroth et al., 2022), and the conclusion gains cross-cultural validity as the phenomenon has also been observed in Japan (Nishimura et al., 2022). The literature concludes that jobs with low control have a higher risk of suicide, which is accentuated by high demand. This explains why low-skilled jobs, with less control over the task, tend to be at higher risk of suicide-related phenomena, both suicidal ideation and attempts (Peek-Asa et al., 2021; Wild et al., 2021; Aberg et al., 2022; Nishimura et al., 2022). It is relevant that when low job control coexists with overqualification of the employee, the risk is accentuated, as it implies a rupture of expectations (Almroth et al., 2022). The study by Nishimura et al. (2022) links the high demand model with objective conditions of precariousness, as he observes in Japan that in highly qualified jobs (white collar), with the ability to control the task performed, the protective effect against the risk of suicide that might exist disappears in the case of overtime.

### 2.2. Dislocation and rupture of times

In the second component of disruption, labor deregulation implies a framework of possible dislocation (Abrutyn, 2019). Labor mobility means the possibility of people losing their ties with the physical spaces they occupy, in a sequence of uprooting with a psychological impact (Fuller, 2011). On the other hand, the poor quality of employment or its absence also generates a limitation in terms of wages, which means the inability to exert power in physical social environments.

The analysis of power over spaces is not an area explored by work psychology, but that of spaces and time frames is. The balance between work time and time dedicated to all other spheres of life is a key mechanism for the construction of solid life projects. This is reflected in the fact that there is solid evidence that work overload is linked to the phenomenon of suicide, both in the form of excessive working hours (Son and Lee, 2021; Wild et al., 2021; Ishikawa, 2022; Kim et al., 2022), and of rotating work shifts and sleep disturbances derived from it (Son and Lee, 2021; Kim et al., 2022). Son and Lee (2021) relate the effect of rotating shifts to stress and suicidal ideation, concluding that the risk of rotating shifts is moderated by the work stress experienced. Gender studies have tended to focus on work-life balance issues, in particular with part-time employment (Wild et al., 2021). Part-time work entails greater precariousness in terms of income, as well as a tendency towards more elementary employment, and affects mainly women, being a cluster of factors clearly related to the phenomenon of suicide (Wild et al., 2021; Kim et al., 2022).

### 2.3. Dissolution: employment, rupture of social bonds and phenomenon of suicide

The third element has to do with dissolution, which relates to the loss of social anchors (Abrutyn, 2019). Returning to the idea of the centrality of employment, work is undergoing a mutation of meaning. Once understood as an element of identity, we assumed that employment generated solid identities that offered a protective factor for the development of social relations in the form of collective identities (Agulló-Tomás, 1997). Labor deregulation prevents the development of these solid identities, leading to the genesis of volatile and liquid identities. This is a psychosocial risk in itself, also generating a relational deterioration (Bauman, 2000; Andrade Jaramillo, 2014).

As can be seen, when analyzing the relationship between the suicidal phenomenon and employment, the most important scientific literature in social psychology is situated on the disjunction axis of Abrutyn’s (2019) model. Fewer studies attempt a more holistic view of the phenomenon of suicide, but there is a line that tries to reflect on the interconnection of phenomena such as social support and work-family balance (Genest et al., 2021), which have to do with bonds, hence with the dimension of dissolution (Abrutyn, 2019). In this sense, Zarate et al. (2022) examines the work sector in the entertainment industry in Australia, which presents a tendency towards isolation. In this context, social support emerges as a highly relevant protective factor against suicidal ideation. However, and in exposing the findings, it is apparent that the research trend does not seek a broad and interconnected understanding of suicide. This is an aspect that we will vindicate with this analytical contribution.

## 3. Discussion: criticisms and future perspectives

Abrutyn’s (2019) model, on which this analysis is based, allows for a coherent and interconnected understanding of suicide, linking occupational, relational and social aspects which, as Rodriguez-Otero et al. (2022) contend, would allow progress to be made towards more efficient and effective preventive models. This responds to a historically claimed priority in the scientific literature on suicide (Goldney, 2003). The ultimate conclusion of this analysis of the state of the issue encourages a change of perspective in labor studies, looking at employment as an integrated and multidimensional element of people’s lives. An analysis that does not allow these different levels of understanding to be interconnected tends to generate a partial and reductionist knowledge about suicide, which does not enable contextualisation in the complexity necessary for the phenomenon studied (Lin et al., 2023). It neglects core factors for suicide such as material conditions, those related to income or housing security, which the literature also indicates are elements of enormous importance for the phenomenon of suicide and intimately linked to the possibilities of employment (Mueller et al., 2021; Peek-Asa et al., 2021). There is also a need to explore the study of racial discrimination, gender, sexual orientation and others in work and non-work contexts in relation to the phenomenon of suicide (Silva and Van Orden, 2018; Sun et al., 2022). There is, therefore, little ability to link social support outside of work, with that which takes place in the work context, and the phenomenon of suicide (Endo et al., 2014). Ultimately, important and relevant evidence is collected to look at employment as a consubstantial element in the understanding of suicide, but studies of employment and suicide lack the holistic view that the phenomenon requires. The reductionism observed highlights an important need to strengthen knowledge about the phenomenon of suicide, opening up a field of study that can be explored in depth on two levels: taking into account the dimensions of disjunction, dislocation and dissolution (Abrutyn, 2019) when understanding work-related phenomena in relation to suicide, and inserting variables of a work-related nature as cross-cutting issues for a complete understanding of the phenomenon of suicide. The psychosocial approach is, therefore, relevant, allowing for the incorporation of a vision and conceptual and theoretical resources that are better suited to offer clarity on this intrinsically complex phenomenon.

## Data availability statement

The original contributions presented in the study are included in the article/supplementary material, further inquiries can be directed to the corresponding author.

## Author contributions

JL: Conceptualization, Writing – original draft. EA-T: Conceptualization, Project administration, Writing – review & editing. SM-E: Conceptualization, Writing – review & editing. BO: Writing – review & editing.
